# Pre-incubation with human umbilical cord derived mesenchymal stem cells-exosomes prevents cisplatin-induced renal tubular epithelial cell injury

**DOI:** 10.18632/aging.103545

**Published:** 2020-09-23

**Authors:** Zongying Li, Shuyi Cao

**Affiliations:** 1 Department of Nephrology, Cangzhou Central Hospital, Cangzhou, Hebei Province, China

**Keywords:** apoptosis, cisplatin, exosomes, renal tubular epithelial cell, viability nephrotoxicity

## Abstract

Purpose: The administration of cisplatin is limited due to its nephrotoxicity, and prevention of this nephrotoxicity of cisplatin is difficult. Mesenchymal stem cell (MSC)-derived exosomes have been implicated as a novel therapeutic approach for tissue injury.

Results: In vitro, the NRK cells pre-incubated with HUMSC-exosomes increased the Cp-inhibited cell viability, proliferation activity, and the cell proportion in G1-phase and inhibited Cp-induced cell apoptosis. Furthermore, the expression levels of apoptotic marker proteins Bim, Bad, Bax, cleaved caspase-3, and cleaved caspase-9 induced by Cp in the NRK cells were decreased by pre-incubating with HUMSC-exosomes.

Conclusion: Our findings indicated that the exosomes from HUMSCs can effectively increase the survival rate and inhibit cell apoptosis of NRK cells. Therefore, pre-treatment of HUMSC-exosomes may be a new method to improve the therapeutic effect of cisplatin.

Patients and methods: Exosomes were isolated from human umbilical cord derived mesenchymal stem cells (HUMSCs). Co-culture of normal rat renal tubular epithelial cells (NRK) and the absorption of exogenous exosomes by NRK cells were examined in vitro. Then the NRK cells were incubated with exosomes from HUMSCs and cisplatin (Cp). Cells were harvested for MTT assay, cloning formation, flow cytometry, and Western blot.

## INTRODUCTION

Kidney is the main metabolic and excretory organ of drugs, and is also the main target of drug toxicity. It has been reported that 34.2% of acute renal failure and 20 % of end-stage kidney disease are caused by side effects of drugs, respectively [1]. Cisplatin (Cp) is an inorganic platinum-based chemotherapy drug. At present, Cp is widely used in the treatment of various malignant tumors, including malignant tumors of the head and neck, lung cancer, ovarian cancer, testicular cancer, bladder cancer [2], and its efficacy is proportional to the dose [3]. However, limiting its full clinical potential is that it has various significant side effects, such as myelosuppression, peripheral neuropathy, ototoxicity, allergic reactions, nephrotoxicity, etc., of which nephrotoxicity is its most serious side effect [4]. Clinically, a single dose of cisplatin (50~100 mg/m^2^) causes the incidence of nephrotoxicity to be as high as 1/3 [5]. Therefore, the prevention and treatment of nephrotoxicity during chemotherapy is one of the urgent problems to be solved in clinical practice.

Mesenchymal stem cells (MSCs) are pluripotent stem cells derived from mesoderm and have the ability to multi-directionally differentiate into fat, osteogenesis, cartilage and neuron, which are present in various tissues and organs of the body [6]. MSCs can homing to the injury site in vivo and promote tissue damage repair by differentiation into damaged cells and paracrine pathways [7]. As an important substance in the secretion of paracrine cells, exosomes play a significant role in promoting cartilage regeneration, reducing ischemia-reperfusion injury and liver and kidney damage [8–11]. Exosomes are biologically active extracellular vesicles secreted by living cells, ranging in size from 30 to 200 nm, which contain abundant biologically active substances such as miRNA, mRNA, and protein [12]. Therefore, exosomes can participate in the regulation of many life activities, and play a greater role in information transmission, disease diagnosis and treatment [13, 14]. Studies have shown that exosomes can effectively reduce the nephrotoxicity of glycerol, gentamicin and cyclosporine by inhibiting oxidative stress [15, 16]. The research implies that exosomes from MSCs may be a novel stem cell-based therapy for kidney diseases.

Therefore, exosomes may be able to reduce Cp-induced kidney damage. In this work, we selected HUMSC-derived exosomes, which confirmed that it can protect and prevent Cp-induced kidney damage. Furthermore, we investigated the protective mechanism of Cp-induced renal tubular cytotoxicity by exosomes, and provided experimental evidence for further clinical research.

## RESULTS

### Characterization and identification of exosomes derived from HUMSCs

**Figure 1:**
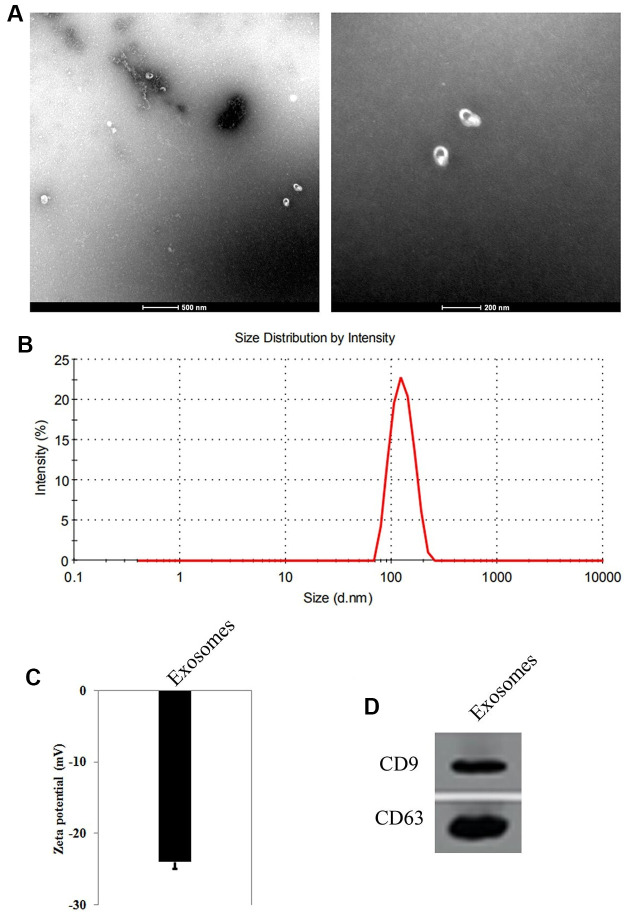
**Identification of exosomes from Human umbilical cord derived mesenchymal stem cells (HUMSCs).** (**A**) Observation of the shape and size of exosomes by transmission electron microscopy. (**B**) Measurement of the size of exosomes by dynamic light scattering. (**C**) Zeta potential of the exosomes. (**D**) The expression of CD9 and CD63, the surface markers of exosomes by Western blot.

The particle size parameters of the exosomes were measured by TEM and DLS. The results showed that the exosomes were microvesicles with a diameter of 80 to 110 nm (Figure 1A), and the peak size of the particle size distribution was 103 nm (Figure 1B). The zeta potential of the exosomes was -24.15±3.1 mV (Figure 1C). Western blot results showed that the exosomes surface markers CD9 and CD63 were highly expressed (Figure 1D). These results suggest that HUMSC-exosomes were successfully separated.

### The exosomes were taken up by NRK cells

**Figure 2:**
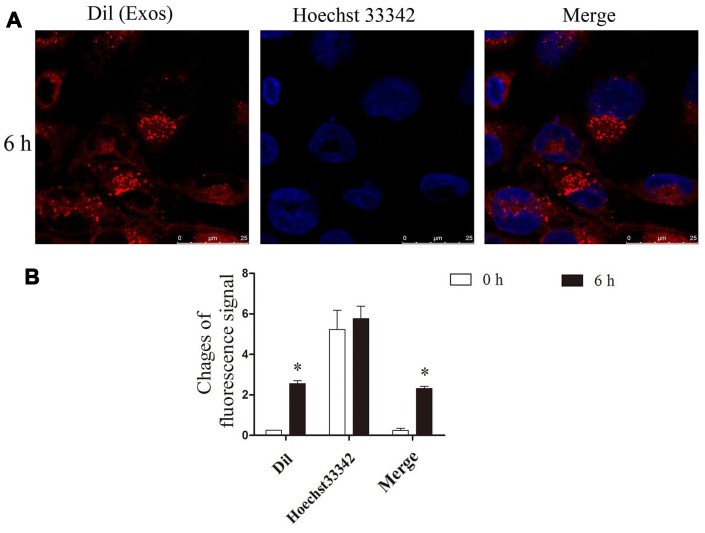
**He exosomes were taken up by NRK.** (**A**) After NRK co-cultured with exosomes, the location of Dil (red), the marker of exosomes, and nucleus (blue) were observed by confocal microscopy. (**B**) The changes of fluorescence intensity of Dil were analyzed by ImageJ.

NRK cells and the exosomes were co-cultured for 6 h. The confocal microscopy observation results showed that Dil-labelled exosomes (red) were taken up by NRK cells, successfully (Figure 2A, 2B).

### Exosomes protect against the injury of NRK cells induced by Cp

**Figure 3:**
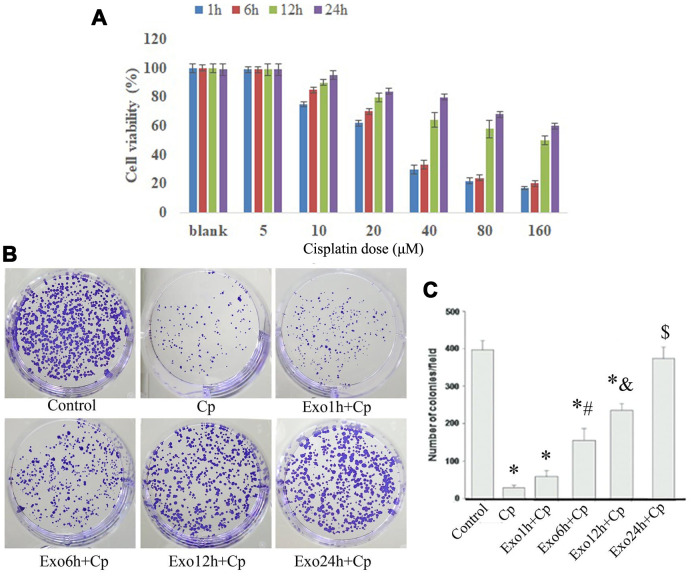
**Cell viability and colony formation detection.** (**A**) After NRK co-cultured with exosomes and Cp, the percentage of cell viability was measured by MTT assay; *, p < 0.05, **, p < 0.01, ***, p < 0.001. (**B**) and (**C**) The changes of colony number were measured by colony formation assay. *, p < 0.05 vs. Control; #, p < 0.05 vs. Exo1h+Cp; &, p < 0.05 vs. Exo6h+Cp; $, p < 0.05 vs. Exo12h+Cp.

The viability of NRK cells co-cultured with exosomes were tested after treating with 5 μM, 10 μM, 20 μM, 40 μM, 80 μM, and 160 μM of Cp, respectively. The MTT results illustrated that NRK cells co-cultured with exosomes for 12 h and 24 h facilitated the viability, while which was inhibited by 10 μM, 20 μM, 40 μM, 80 μM, and 160 μM of Cp (Figure 3A). The colony formation of NRK cells was tested after treating with Cp, Exo1h+Cp, Exo6h+Cp, Exo12h+Cp, and Exo24h+Cp, respectively. Compared with the Control group, the number of cell colony in the Cp group was decreased. However, relative to the Cp group, the number of cell colony was upregulated in the Exo6h+Cp, Exo12h+Cp, and Exo24h+Cp groups (Figure 3B, 3C), indicating that HUMSC-exosomes relieved Cp-induced injury of NRK cells.

### Exosomes attenuates Cp induced NRK cells apoptosis

**Figure 4:**
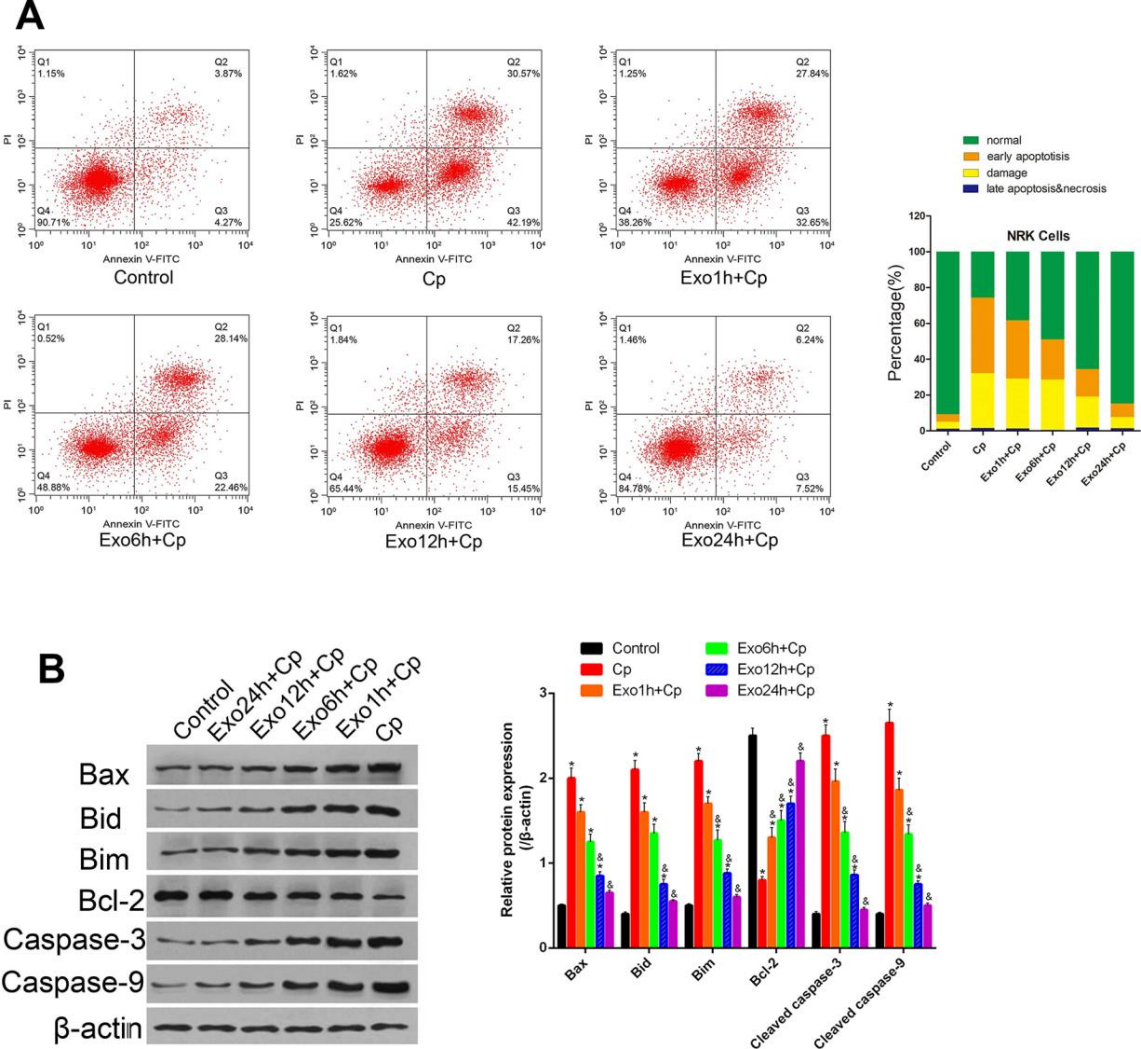
**Effect of different groups on NRK apoptosis.** (**A**) Apoptosis was tested by Annexin V-FITC/PI staining method. (**B**) The protein levels of Bax, Bid, Bim, Bcl-2, cleaved caspase-3, and cleaved caspase-9 were measured by Western blot, and analyzed by ImageJ. * P< 0.05 vs. Control; &, p < 0.05 vs. Exo1h+Cp.

The apoptosis of NRK cells was tested after treating with Exo1h+Cp, Exo6h+Cp, Exo12h+Cp, and Exo24h+Cp. Compared with the Control group, the apoptosis levels of the Cp group were upregulated (Figure 4A). However, relative to the Cp group, the apoptosis levels were downregulated in the Exo1h+Cp, Exo6h+Cp, Exo12h+Cp, and Exo24h+Cp groups (Figure 4A).

### Exosomes regulates the expression of apoptotic marker proteins in NRK cells treated with Cp

To further clarify the protective mechanism of HUMSC-exosomes on apoptosis of kidney cells, the expression levels of apoptotic marker proteins Bax, Bid, Bim, Bcl-2, cleaved caspase-3, and cleaved caspase-9 were measured by Western blot. Compared with the Control group, the level of Bcl-2 was decreased and the levels of Bax, Bid, Bim, cleaved caspase-3, and cleaved caspase-9 were upregulated in the Cp group (Figure 4B). However, relative to the Cp group, the levels of Bcl-2 were upregulated and the levels of Bax, Bid, Bim, cleaved caspase-3, and cleaved caspase-9 were downregulated in the Cp group in the Exo1h+Cp, Exo6h+Cp, Exo12h+Cp, and Exo24h+Cp groups (Figure 4B). It was illustrated that HUMSC-exosomes inhibited the NRK cells apoptosis induced by Cp.

### Exosomes regulates the cell cycle progression of NRK cells

**Figure 5:**
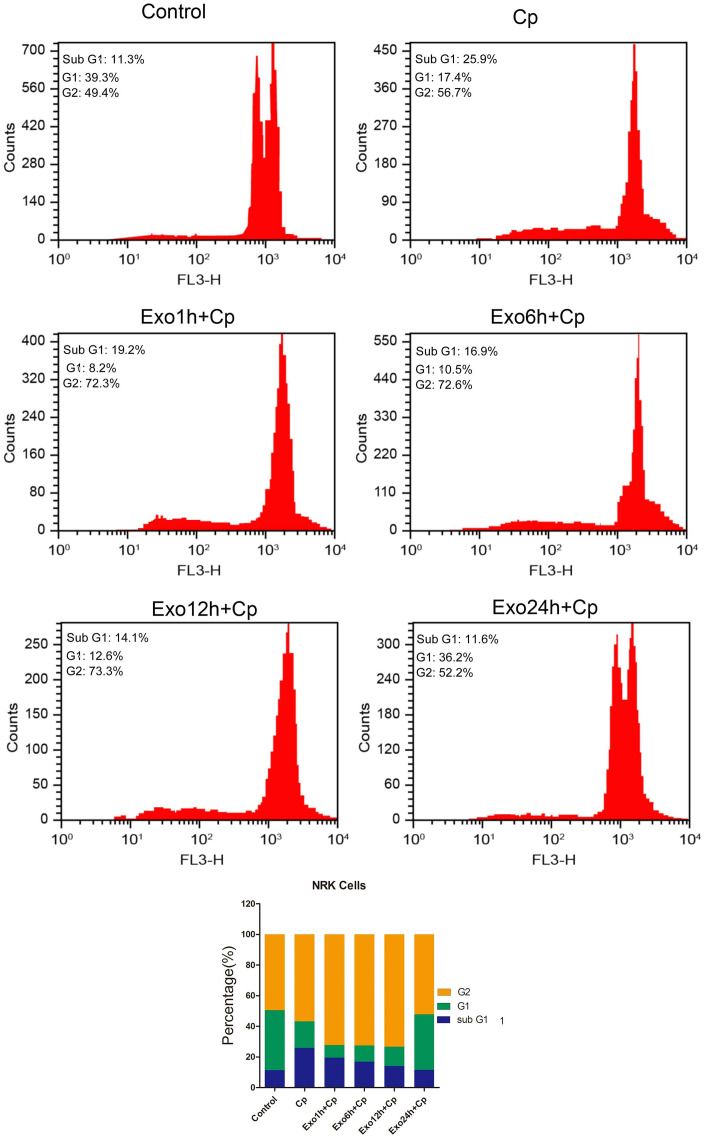
**Effect of different groups on NRK cell cycle progression.** The cell cycle progression was tested by flow cytometry.

The changes of cell cycle progression were measured after treating with Exo1h+Cp, Exo6h+Cp, Exo12h+Cp, and Exo24h+Cp. Compared with the Control group, the proportion of the cells in G1-phase in the Cp group was decreased (Figure 5) and that of the cells in the G2-phase was increased (Figure 5). However, relative to the Cp group, the proportion of the cells in G1-phase in the Exo24h+Cp group was increased (Figure 5), suggesting that HUMSC-exosomes could improve cycle progression of NRK cells.

## DISCUSSION

Cp is a potent anticancer agent and its mechanism of action may be related to its high intracellular reactants. In an aqueous environment in a cell, Cp forms a conjugate with intracellular glutathione, protein, RNA, and DNA, and crosslinks with the purine base of DNA, interfering with DNA repair mechanisms, leading to DNA damage, further induction. Thus, it induces cancer cells to be apoptotic, but they are also toxic to normal cells [17]. Cp and its hydrated or hydroxyl metabolites are excreted primarily through the kidneys. Because of low molecular weight and no charge of Cp, the free Cp in plasma is easily filtered by the glomerulus, which results in a concentration of 5 times that in the proximal tubular epithelial cells. It is consequently more sensitive to the toxic effects of Cp, and the nephrotoxicity of Cp is most serious. However, the nephrotoxicity caused by Cp is currently lacking effective prevention and treatment methods [18]. In the present study, Cp with gradually elevated concentration decreased the viability and colony formation of the NRK with dose-dependent. It was indicated that Cp was highly toxic to the NRK. Furthermore, studies have shown that Cp damages proximal tubular epithelial cells, leading to apoptosis of renal tubular cells mainly involved in mitochondria-mediated endogenous pathways, death receptor-mediated exogenous pathways and endoplasmic reticulum stress pathways [19]. We also verified the induction effect of Cp on NRK apoptosis in the present study, which was consistent with previous research results.

In recent years, as the main biological component of paracrine secretion of stem cells, exosomes reduce the possibility of stem cells forming tumors *in vivo* because they do not have the ability to divide and differentiate [20, 21]. Compared with mesenchymal stem cell transplantation therapy, exosomes have relative stability. Exosomes are distinguished by their size from other vesicles secreted by mesenchymal stem cells, ranging from 30-200 nm in diameter to densities ranging from 1.10 to 1.20 g/mL [12]. The key step in the present study is the extraction of exosomes. The present study used the low temperature ultra-high speed centrifugation method to obtain relatively pure exosomes. Under electron microscope, the exosomes were mostly concentrated at 80-110 nm, and can express CD9 and CD63 marker surface proteins. Consistent with the biological characteristics and identification criteria of exosomes, it indicated that the exosomes of HUMSCs were successfully isolated. Furthermore, in pig and mouse models of myocardial ischemia-reperfusion injury in mice, Timmers et al. [22] found that the medium reduced myocardial infarct size by 60% and 50%, respectively, when intravenously injected into conditioned medium of mesenchymal stem cells. It was further confirmed that the size of the active medium acting was in the range of 50-200 nm. The study found that mesenchymal stem cell conditioned medium can reduce myocardial infarct size in mice, but conditioned medium without exosomes does not have this function [23]. All of the above studies have demonstrated that mesenchymal stem cells mainly function through exosomes in their supernatants. In the present study, notably, when exosomes were co-cultured with Cp-treated NRK, the cell viability of NRK was remarkably higher than that of the Cp-treatment alone. In addition, by increasing the culture time of exosomes and NRK, it was found that the proliferative capacity of NRK was positively correlated with the culture time of exosomes. These results suggested that exosomes from HUMSCs promoted renal endothelial cell proliferation.

In order to explore the protection of the exosomes on Cp-induced NRK cell injury, its effect on apoptosis was observed. The results showed that the apoptosis rate of NRK cells was up-regulated after Cp treatment, and the expression of apoptosis markers Bax, Bid, Bim, Caspas-3 and -9 were up-regulated. Notably, after the exosomes were added to the Cp-induced NR, the apoptotic rate increased significantly, and the expressions of Bax, Bid, Bim, and Caspas-3 and -9 were all down-regulated, and the expression of Bcl-2 was upregulated. It was indicated that exosomes promoted the proliferation of NRK cells and inhibit the apoptosis of cells, which consistent with previous results in exosomes regulating apoptosis and proliferation of endothelial cells [24]. Our further results showed that the cells incubated with exosomes alleviated cell cycle inhibition caused by Cp. In recent years, a large number of studies have shown that exosome miRNAs play an important role in the occurrence and development of diseases. Exosomes can selectively encapsulate miRNAs and stably transfer miRNAs to recipient cells and function [25]. Additionally, in diseases such as lung cancer, lung inflammation, and pulmonary fibrosis, exosome miRNAs regulate the expression of many proliferation-related and/or apoptosis-related genes [25–27]. For example, cardiac stem cell-derived exosomes miR-21 inhibited cardiomyocyte apoptosis by targeting binding to programmed cell death factor 4 [28]. We hypothesized that the protective effect of exosomes on NRK cells may be related to the regulation of cell proliferation and apoptosis-related genes by the miRNAs they carry. However, this speculation still needs further experimental verification.

The clinical application of exosomes depends on the technical breakthrough of exosome-based drug delivery system. At present, exosomes have been found to play an important role in various health and disease models through the transmission of molecular information. Exosomes are recognized as biomarkers and prognostic factors of diseases and have important clinical diagnostic and therapeutic significance. In addition, they have the potential to be used clinically as a vehicle for gene and drug delivery. Exosomes themselves are quite inert, but when they fuse with the cell membrane, they can deliver the materials and signals they carry to the recipient cell and change its biological function. Therefore, exosomes are potential carriers for nanometer drug delivery or gene therapy. As natural carriers of functional small RNAs and proteins, exosomes can be used to deliver various ribonucleic acid molecules, peptides and synthetic drugs in the field of drug delivery.

In this study, the functionally exosomal miRNAs have not been screened and explored. In the later stage, we will screen for miRNAs in the process of regulating the function of renal tubular epithelial cells by miRNA microarray or high throughput sequencing technology. In summary, exosomes from HUMSCs can significantly reduce Cp-induced NRK cell injury, which may be related to the up-regulation of Bcl-2 in renal tubular cells and inhibition of Bax, Bid, and Bim expressions, thereby inhibiting downstream caspase-3 and caspase-9 activation. Exosomes may be an ideal Cp nephrotoxicity control drug and deserve further study. And these findings provide a basis for the future use of exosomes as a new biological therapeutic approach for renal diseases and injuries.

## CONCLUSION

In this study, the functionally exosomal miRNAs have not been screened and explored. In the later stage, we will screen for miRNAs in the process of regulating the function of renal tubular epithelial cells by miRNA microarray or high throughput sequencing technology. In summary, exosomes from HUMSCs can significantly reduce Cp-induced NRK cell injury, which may be related to the up-regulation of Bcl-2 in renal tubular cells and inhibition of Bax, Bid, and Bim expressions, thereby inhibiting downstream caspase-3 and caspase-9 activation. Exosomes may be an ideal Cp nephrotoxicity control drug and deserve further study. And these findings provide a basis for the future use of exosomes as a new biological therapeutic approach for renal diseases and injuries.

## MATERIALS AND METHODS

### Cell culture

HUMSCs were purchased from Cyagen Biosciences (Guangzhou, China). HUMSCs were culture in DMEM containing 10% fetal bovine serum at 37 °C and in 5% CO_2_ incubator. Furthermore, normal rat renal tubular epithelial cells (NRK) were purchased from Procell (Wuhan, China) and were cultured in DMEM (PM150210) containing 10% fetal bovine serum at 37 °C and in 5% CO_2_ incubator.

### Exosomes separation

The culture supernatant of HUMSCs in 4-6 generations was collected and the exosomes were extracted according to the instructions of the Invitrogen 4478359 Total Exosome Isolation Reagent (Gibco, Invitrogen, UK). Briefly, the culture supernatant was transferred to a high-speed centrifuge tube, centrifuge at 4°C, 2000 g for 30 min, the supernatant was taken and transferred to a new high-speed centrifuge tube, and the cell supernatant was added (cell supernatant: reagent = 2:1). After fully mixing with vortex, the mixture was placed in a refrigerator and incubated at 4 °C overnight. After centrifugation at 4 °C, 10000 g for 60 min, the precipitate was resuspended in 100 μl of cold PBS as a suspension of exosomes and stored at -80 °C.

### Identification of exosomes

Transmission electron microscope (TEM) was used to observe the morphology of exosomes. 10 μl of the exosomes were separated and purified, and diluted with an equal volume of balanced salt PBS solution. The sample was added dropwise to a sample-loaded copper mesh, and was allowed to stand at room temperature for 1 minute. Then the excess liquid was gently removed using filter paper. After that, the sample was negatively stained with 3% sodium phosphotungstate solution (pH 6.8) for 5 minutes at RT. After gently washing with double distilled water, the sample was air-dried at room temperature, and observed under a transmission electron microscope. In addition, dynamic light scattering (DLS) was used to detect the size of exosomes. Briefly, 10 μl of the exosomes were separated and purified, and diluted with PBS solution to measure the size using a Malvern laser particle size analyzer.

### Western blot

Western Blot was used to identify the expressions of CD9 and CD63 on the surface of exosomes and the expressions of Bax, Bid, Bim, Bcl-2, cleaved caspase-3, and cleaved caspase-9 in NRK cells. Total proteins were obtained using lysis buffer, and then quantitated by bicinchoninic acid kit (Beyotime, Shanghai, China). Following sample separating and transferring into PVDF membranes, membranes were immerged in 5% nonfat milk. Next, primary antibodies of CD9, CD6, Bax, Bid, Bim, Bcl-2, cleaved caspase-3, cleaved caspase-9 (1: 800, Abcam), and β-actin (1: 1000, Beyotime), respectively, were used for immunoblotting of the membranes overnight at 4°C. After the incubation with second antibody (1:5000, Jackson, USA), the protein levels were detected by enhanced chemiluminescence (ECL, Millipore, USA).

### Observation of cell uptake by laser confocal microscopy

Dil (Thermo Fisher Scientific, Invitrogen, catalog number: D282) was used to mark the exosomes. After staining for 30 min, the exosomes were washed with PBS and collected by centrifugation at 10000 g for 60 min to obtain the Dil-labeled exosomes. NRK cells were planted in confocal dishes. After the cells were attached overnight, 20 μg/ml of Dil-labeled exosomes were added to the dish, and after 1, 4, 8, 12, and 24 h of culture, the cells were washed with PBS and fixed with 4% paraformaldehyde for 30 min. Subsequently, after washing with PBS, the cells were permeabilized for 15 min using 0.2% Triton-100. Nucleus were stained with Hoechst 33342, and photographed using laser confocal after washing with PBS.

### Cell viability by MTT (thiazolyl blue tetrazolium bromide)

NRK cells were pre-incubated with exosomes from HUMSCs for 1 h, 6 h, 12 h, and 24h, followed by treatment with Cp at 5, 10, 20, 40, 80, and 160 μM in 96-well plates. After co-culturing 24h, cell viability was measured using the MTT assay. MTT solution (5 g/L) was added at 10 μL/well. After incubating for 4 h, the supernatant was removed, 150 μL of DMSO was added to each well, and the cells were gently shaken for 30 s. 100 μL of each well was aspirated into the enzyme label, and then the absorbance value (OD) at 570 nm was measured by a microplate reader. The percentage of cell activity was used as an evaluation index for the effect of nanoparticles on cell viability. The calculation formula was: percentage of cell activity (%) = (OD value of each well in the infected group - blank OD value) / (Control group OD mean - blank OD value) × 100%, control group cell activity percentage was 100%.

### Colony formation assay

NRK cells in the Control, Cp, Exo1h+Cp, Exo6h+Cp, Exo12h+Cp, and Exo24h+Cp. groups were respectively mixed with 0.3% Noble agar in 10% FBS supplemented DMEM, which were seeded as the upper agar layer of the agar plates. The cells were then incubated in a 37 °C incubator for 7 days, and the number of colonies was counted.

### Cell apoptosis by flow cytometry

NRK cells were seeded in 6-well plates overnight, 200 μg/ml of exosomes was added. After co-culturing for 1 h, 6 h, 12 h, and 24 h, 20 μM Cp was added in to the plates. The NRK cells were divided into six groups: no treatment group (Control), Cp group, co-culture with exosomes combined with Cp treatment groups for 1 h (Exo1h+Cp), 6 h (Exo6h+Cp), 12 h (Exo12h+Cp), and 24 h (Exo24h+Cp). After Cp treatment for 24 hours, cell apoptosis was detected by flow cytometry using Annexin-PI staining.

### Cell cycle detection by flow cytometry

The NRK cells were seeded in 6-well plates overnight were pre-incubated with exosomes from HUMSCs for 1 h, 6 h, 12 h, and 24h, followed by treatment with 20 μM Cp which prepared by a medium containing 200 μg/ml of exosomes. Then, the cells in each group were collected. 5X10^4^ cells in each group were washed twice with PBS, and fixed in 1 mL of 70% ice ethanol for 24 h at 4 °C. Then, the cells were washed twice with PBS and were added with propidium iodide staining solution at 4 °C for 30 min. Cell cycle distribution was measured using a flow cytometer.

### Statistical analysis

GraphPad Prism 6.05 was used for statistical analysis of experimental data. One-way ANOVA and Student's t test were used for comparative analysis of differences. *P* < 0.05 was considered statistically significant.
